# Assessing meiofaunal variation among individuals utilising morphological and molecular approaches: an example using the Tardigrada

**DOI:** 10.1186/1472-6785-8-7

**Published:** 2008-04-30

**Authors:** Chester J Sands, Peter Convey, Katrin Linse, Sandra J McInnes

**Affiliations:** 1 British Antarctic Survey, Natural Environment Research Council, High Cross, Madingley Road, Cambridge, CB3 0ET, UK

## Abstract

**Background:**

Meiofauna – multicellular animals captured between sieve size 45 μm and 1000 μm – are a fundamental component of terrestrial, and marine benthic ecosystems, forming an integral element of food webs, and playing a critical roll in nutrient recycling. Most phyla have meiofaunal representatives and studies of these taxa impact on a wide variety of sub-disciplines as well as having social and economic implications. However, studies of variation in meiofauna are presented with several important challenges. Isolating individuals from a sample substrate is a time consuming process, and identification requires increasingly scarce taxonomic expertise. Finding suitable morphological characters in many of these organisms is often difficult even for experts. Molecular markers are extremely useful for identifying variation in morphologically conserved organisms. However, for many species markers need to be developed *de novo*, while DNA can often only be extracted from pooled samples in order to obtain sufficient quantity and quality. Importantly, multiple independent markers are required to reconcile gene evolution with species evolution. In this primarily methodological paper we provide a proof of principle of a novel and effective protocol for the isolation of meiofauna from an environmental sample. We also go on to illustrate examples of the implications arising from subsequent screening for genetic variation at the level of the individual using ribosomal, mitochondrial and single copy nuclear markers.

**Results:**

To isolate individual tardigrades from their habitat substrate we used a non-toxic density gradient media that did not interfere with downstream biochemical processes. Using a simple DNA release technique and nested polymerase chain reaction with universal primers we were able amplify multi-copy and, to some extent, single copy genes from individual tardigrades. Maximum likelihood trees from ribosomal 18S, mitochondrial *cytochrome oxidase subunit *1, and the single copy nuclear gene *Wingless *support a recent study indicating that the family Hypsibiidae is a non-monophyletic group. From these sequences we were able to detect variation between individuals at each locus that allowed us to identify the presence of cryptic taxa that would otherwise have been overlooked.

**Conclusion:**

Molecular results obtained from individuals, rather than pooled samples, are a prerequisite to enable levels of variation to be placed into context. In this study we have provided a proof of principle of this approach for meiofaunal tardigrades, an important group of soil biota previously not considered amenable to such studies, thereby paving the way for more comprehensive phylogenetic studies using multiple nuclear markers, and population genetic studies.

## Background

Assessing variation within and between species provides information relating to taxonomic relationships [1,2], as well as population structure, demographics and biogeographic patterns [3,4]. Microscopic animals that are collectively known as the meiofauna present a series of challenges to studies of variation. Despite their small size (generally captured in sieve mesh sizes between 45 μm and 1000 μm), meiofauna form an integral and vital component of the soil food web, playing a fundamental role in soil ecosystem processes, particularly in nutrient recycling and decomposition processes. Twenty of the 34 recognised animal phyla have meiofaunal representatives, 5 of which are exclusively meiofaunal [5], the implication being that in a handful of soil or sediment there is likely to be a high level of biodiversity. In more extreme environments, such as those of the Antarctic, meiofauna may constitute the majority of, and in some cases the only, metoazoan element present in the soil ecosystem [6-8]. Meiofauna are a focus of research from many sub-disciplines (including epidemiology, ecology, soil science, agriculture, aquaculture, and pollution monitoring), as well as providing model organisms for studies of evolutionary development (the nematode *Caenorhabditis elegans*) and the evolution of sex (bdelloid rotifers). These tiny animals are difficult to see, often smaller than the particulate matter that comprises the substrata they inhabit, and thus difficult to separate from sediment, detritus and non-target species. Within taxonomic groupings there may be few visible distinguishing characters, and those that are present may be subtle, requiring specialist and increasingly scarce taxonomic expertise [9]. Nevertheless, accurate species identification is an essential first step to any scientific study.

Molecular phylogenetic techniques take advantage of developments in our ability to detect variation in DNA, effectively increasing the resolution available in comparison with morphological or phenotypic variation. This can be particularly useful when dealing with morphologically conserved groups. Each base change in a DNA sequence is analogous to a distinct morphological character in a phylogenetic analysis, which means many hundreds of characters can potentially be included in a single gene analysis. Models have been developed to take rates of evolution and homoplasy into account [10,11] providing molecular phylogenetic inference with a robust theoretical basis [12]. The concept that variation in a suitable and defined length of DNA sequence may be used as a bar code for species identification is becoming increasingly appreciated [13,14].

An important advantage of molecular data is that independent replication is possible. A phylogeny based on a single gene provides information regarding the history of and relationships between the taxa sampled. However, the information is limited to the occurrence of mutations and results in what is known as the genetree/species tree problem [15]. A significant event in the history of a lineage will only be recorded in a phylogeny if a mutation occurs at or just after the event. Conversely a high mutation rate may lead to a confused signal in cases where, for instance, an informative mutation reverts back to its ancestral state, or further changes to a state present in another lineage (homoplasy). Furthermore, there are processes, such as selection, that may affect the evolution of a gene, thereby confusing the signal of evolutionary history of the organism. These caveats may be at least partly countered by conducting independent analyses on several unlinked genes in order to generate a general consensus of phylogenies that more closely reflects the "true" evolutionary history, or by concatenating sequences to produce a super phylogeny [16].

It is often not appreciated that transferring molecular techniques from the few "model" organisms that are the focus of intense laboratory research to the more numerous but less studied groups is not straightforward. Conversely, molecular ecologists who work on meiofauna and other invertebrates may be surprised when they find their difficulties are not appreciated by the wider scientific community. DNA quantity is a major issue with tiny organisms. To increase DNA yield, the whole organism or, more often several pooled individuals, are used in extractions and, thus, problems with contaminants from gut contents or commensal organisms arise [17,18]. The difficulties working with understudied species are often only evidenced by the lack of published literature available. For example, as far as we are aware, there have only been three assessments made of intraspecific variation in any species of the meiofaunal phylum Tardigrada [19-21]. This may be partially due to tardigrades and other meiofaunal groups falling outside economic and charismatic categories that influence the direction of science programs, but it is also likely to be due to the difficulties involved in obtaining genetic data from these members of the meiofaunal community. Recently a variety of techniques have been developed or demonstrated as suitable for DNA extraction from a variety of meiofauna [2,22-25] providing a basis for basic evolutionary studies and the development of molecular tools for identification or classification [20,22,23,26].

Tardigrades are found in most terrestrial, freshwater and marine habitats, including some remote Antarctic nunataks where they are found in the absence of the otherwise ubiquitous nematodes [6]. They are one of the few phyla with representatives found from the highest and coldest terrestrial environments to the deepest oceans. They have featured in high impact publications mostly due to their uncertain phylogenetic position in the tree of life [27-30], although their ubiquitous distribution makes them ideal candidates for historical biogeographic reconstruction [31-33]. Molecular phylogenetic work to date is strongly concordant with morphological based systematics [29], supporting both tardigrade monophyly and monophyly in the constituent classes Eutardigrada and Heterotardigrada [17,24,34].

In this paper we describe a method that enables the quantitative and qualitative assessment of morphology and genetic variation among individual tardigrades. The method includes a novel technique for separating all organisms from their substrate as compared with "cherry-picking" visible organisms under a dissecting microscope, a simple DNA release technique applicable at the individual level, and a general protocol for amplifying genomic DNA from multicopy and single copy genes. Previous studies have amplified multi-copy 18S and high-copy number (CO1) genes from tardigrades [2,20,25,26], but this is the first time single copy nuclear genes have been amplified from genomic DNA extracted from a single tardigrade. We demonstrate that this method allows for informed re-assessment of morphological variation, allows independent replication of phylogenetic analyses and is suitable for assessing within population variation enabling population genetic studies.

## Results and Discussion

### Sample collection and preparation

Separating meiofauna from the substrate can be done in two ways: "cherry-picking" individuals using a pipette or Irwin loop under a microscope, or mechanically separation, usually using a density gradient. "Cherry-picking" is a fast technique if only a few specifically targeted individuals are required and the organisms are easily identifiable from the substrate. However, if quantitative biomass and diversity data are required this technique will be biased towards larger, vagile and more or less pigmented organisms (depending upon which contrasts with the substrate). Where quantitative and qualitative results are required to extract all individuals from a substrate the cherry-picking approach is inappropriate and such studies have adopted mechanical separation using density gradients [see [35]]. However, the media used, such as Ludox AM (Dupont, France) [35-37], Percoll (Pharmacia, Uppsala, Sweden) [38] or 50% sucrose [39] may have detrimental downstream effects, particularly for PCR. Using OptiPrep™ – Density Gradient Media (Axis-Shield, UK) we were able to isolate and identify all individual tardigrades and eggs from habitat substrata (see methods). Substrata used included fresh, frozen and dried herbarium moss specimens. Contrasting with previous protocols OptiPrep is non-ionic, non-toxic and does not require washing to remove the media. It has been used to fractionate sub-cellular organelles and does not interfere with marker enzyme activities, allowing fractions of cell organelles to be analyzed without removal of the gradient medium [40]. In the current study it proved to be clean and efficient at extracting meiofauna from substrata, allowing biodiversity and biomass from each sample to be determined, and detailed morphological examination without affecting subsequent DNA extraction or downstream biochemical processes. Furthermore, tardigrades extracted from fresh and frozen samples remained alive after the extraction process.

### DNA extraction

Taking into account the limited amount of DNA available in a single tardigrade we opted for a DNA release method rather than an extraction method to maximise the amount of DNA available. We trialled several different release methods (NaOH digestion, [22]; TE/ddH_2_O boil, [41]; Proteinase K freeze thaw cycles, [23]). All these techniques were sufficient for amplification of 18S rDNA, as many more expensive commercial kits have proven to be [24]. However, we found a 20 min boil in 40 μL of 5% chelex the most reliable technique across all gene regions, possibly because the chelex beads inactivate inhibitors that would otherwise prevent reliable amplification. Obviously the risk of contamination and competition in PCR is significant and all precautions are advised to prevent inadvertent introduction of foreign DNA. Fortuitously this technique is cheap and rapid and the only reagent necessary (Chelex 100 – BIORAD) is inexpensive and readily available in many molecular laboratories.

### DNA quantification

In order to quantify DNA concentration we used a PicoGreen™ assay (Molecular Probes), a sensitive technique for determining concentrations of DNA in a solution. After extensive optimization to reduce the volumes of template required and level of background fluorescence, we were unable to detect DNA in any of our extractions. However, we were able to successfully amplify ribosomal *18S *and mitochondrial *cytochrome c oxidase subunit *1 (*CO1*) products from most of our tardigrade extractions – even from animals extracted from rehydrated herbarium samples that had been stored dry for 15 years. Recently Kiel et al [42] extracted 8.4 μg of genomic DNA from ~2000 pooled *Hypsibius klebelsbergi*, indicating that each tardigrade contributed ~4.2 ng of DNA to the extract. For amplification using polymerase chain reaction, 20 – 50 ng of DNA is usually recommended in a 1 μL volume. Clearly this will never be possible at individual level for many meiofaunal groups – particularly tardigrades – making studies of individuals rather than pooled samples challenging. Even with the advent of whole genome amplification, to achieve representative amplification of the genome a minimum starting quantity of 1 μl of 10 ng/μL is recommended (GE HealthCare: GenomiPhi™ instruction manual).

### Polymerase Chain Reaction (PCR)

Our strategy for amplifying genomic DNA was to use nested or hemi-nested PCR (see Table 1 for primer sequences). Rarely was product visible on a gel after a single round of PCR. However, re-amplifying from the first reaction using at least one internal primer resulted in reasonable amplification of the desired product. Large gene regions (e.g. *18S*) were amplified in overlapping fragments; first the whole region was amplified, then using this reaction mix as template three overlapping fragments were amplified. Products amplified in this way generally returned clean sequences. Two of the three *18S *fragments amplified reliably on all templates, fragment one was difficult to amplify in heterotardigrades, while *CO1 *was less reliable and *Wingless *amplified only template from fresh or recently frozen samples. We were also able to amplify *Alpha Spectrin *(*aspec*) *and Elongation Factor 1 alpha *(*EF1*a) from some individuals. We were able to verify that the *aspec *sequences we obtained were homologues but we were unable to verify these as originating from Tardigrada. We were able to obtain *EF1a *sequences from most individuals, however, up to 8 different paralogues were identified, each with remarkably conserved coding sequence making orthologue-specific primer redesign difficult (data not presented). GenBank accession numbers and sample details can be found in the additional information file associated with this paper.

**Table 1:** Primers used in each reaction combination

		Forward	Reverse
**18S**			
Amp 1		SSU01_F	SSU82_R_short
		AACCTGGTTGATCCTGCCAGT	TGATCCTTCTGCAGGTTCACC
Amp 2			
	Fragment 1	SSU01_F	SSU26_R
		AACCTGGTTGATCCTGCCAGT	CATTCTTGGCAAATGCTTTCG
	Fragment 2	SSU22_F	SSU13_R
		TCCAAGGAAGGCAGCAGGC	GGGCATCACAGACCTGTTA
	Fragment 3	SSU26_F	SSU82_R
		CGAAAGCATTTGCCAAGAATG	TGATCCTTCTGCAGGTTCACCTAC

**CO1**			
Amp 1		LCO_1490	mtD9_2206
		GGTCAACAAATCATAAAGATATTGG	CCCGGTAAAATTAAAATATAAACTTC
Amp 2		LCO_1490	HCO_2198
		GGTCAACAAATCATAAAGATATTGG	TAAACTTCAGGGTGACCAAAAAATCA

**Wingless**			
Amp 1		Wg1s_F	Wg2n_R
		GARTGYAARTGYCAYGG	ACYTCRCARCACCARTG
Amp 2		Wg1a_F	Wgs_R
		GARTGYAARTGYCAYGGYATGTCTGG	ACYTCRCARCACCARTGRAA

**Alpha Spectrin**			
Amp 1		Aspec11_F	Aspec15_R
		TGGATHMGNGARAARGA	AARTCRTCRAAYTTYTTYTG
Amp 2		Aspec11_F	Aspec12_R
		TGGATHMGNGARAARGA	ACYTCNACYTTCCACCARTC

**Elongation Factor 1alpha**			
Amp 1		237_F	1450_R
		CGGYCAYTTGATCTACAAATGC	TGTCRCGCACAGCGAAACKACC
Amp 2		277_F	1221L_R
		ACSATYGAGAAGTTCGAGAAG	GGRTGRTTMARIACRATMACCTG

Each sequence corresponds to one of two oligo-nucleotide primers used for each specific reaction. In addition, all forward primers were tailed with M13_REV (CAGGAAACAGCTATGACC) and all reverse primers were tailed with M13 – 21 (TGTAAAACGACGGCCAGT). Primers for *18S *were adapted from [55]. LCO/HCO primers for *CO1 *were designed by [56]. mtD9 was designed by [57]. *Wingless *and *Alpha Spectrin *primers were sourced from [58]. *Elongation Factor 1 alpha *primers were designed from GenBank alignments of onychophora, tardigrade, drosophila and artemia.

### Phylogenetic reconstruction

**Figure 1:**
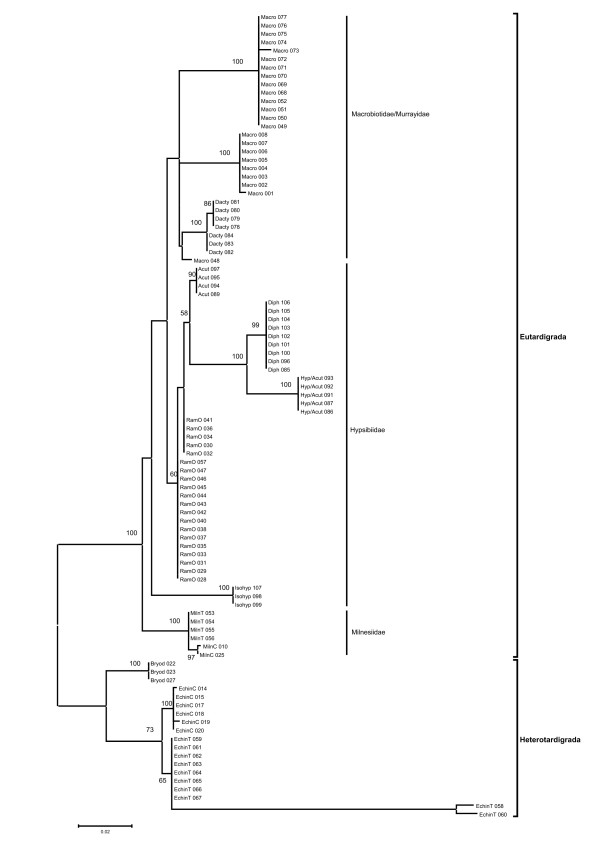
**Tardigrade phylogeny constructed from 18S rRNA**. Phylogeny produced using maximum likelihood analyses under a GTR+I+Γ model using 884 bases of *18S *rRNA. Numbers at nodes are support values generated from 1000 bootstrap pseudoreplicates. Terminal labels associated with unique numbers were identified as: Macro 001–008 – *Macrobiotus furciger*, Macro 048-52,68-077 – *Macrobiotus sp*. (hufelandi type), Dacty – *Dactylobiotus sp*., Acut – *Acutuncus antarcticus*, Diph – *Diphascon sp*., RamO – *Ramazzottius oberhaeuseri*, Isohyp – *Isohypsibius asper*, MilnT – *Milnesium tardigradum*, MilnC – *Milnesium sp. *"charcot", Bryod – *Bryodelphax sp*., EchinC – *Echiniscus sp*., EchinT – *Echiniscus testudo*.

**Figure 2:**
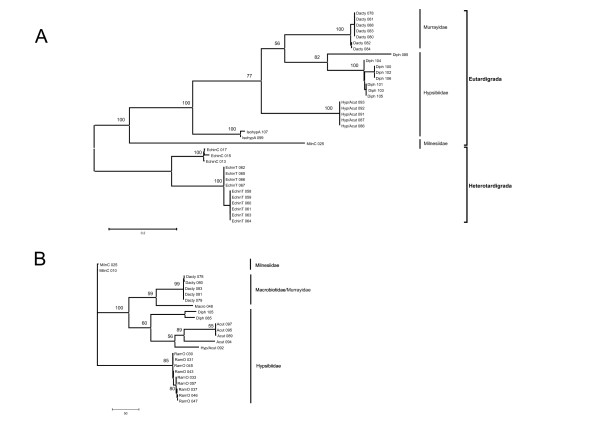
**Tardigrade phylogeny constructed from CO1 and Wingless**. Phylogenies produced using maximum likelihood analyses under GTR+I+Γ models using: A. 534 bases of mitochondrial CO1 and B. 321 bases of Wingless. Terminal labels correspond to those described in Figure 1. Support at nodes was generated using 1000 bootstrap pseudoreplicates.

Using maximum likelihood methods we have constructed phylogenies from nuclear ribosomal *18S*, mitochondrial *CO1 *and nuclear *Wingless *regions (Figures 1 and 2). As we were unable to amplify heterotardigrades using *Wingless *primers we used *Milnesium *sp. as an out-group in this case as it is believed to represent a basal eutardigrade [17,23,28]. For the *18S *and *CO1 *phylogenies we used heterotardigrades as out-groups to root the trees and explore the relationships within the eutardigrades. Although all studies to date have relied on a small number of taxa (11 tardigrade taxa in [17]), results of both molecular and morphological work indicate monophyly of heterotardigrades and eutardigrades, and Order Apochaela holding a basal position within the Eutardigrada [17,23,28]. Our results are consistent with these findings. The most striking feature contained in these phylogenies is the non-monophyletic relationship between the eutardigrade families Hypsibiidae and Macrobiotidae and Murrayidae, with Macrobiotidae and Murrayidae a clade nested within Hypsibiidae. Although not discussed, this is also evident in the phylogeny of Jørgensen and Kristensen [17] and has recently been independently identified by Kiehl et al 07 [34]. By utilizing our current method, further work is planned to fully explore phylogenetic relationships among and within tardigrade families.

### Identifying molecular variation

An important advantage of analysing sequences from individuals rather than a pooled sample is that variation that is difficult to distinguish using morphology is likely to be identified. For example, *Macrobiotus *sp. is clustered into two groups, one containing individuals from near Ville de Jumelles, St Maur Créteil, France, the other containing individuals from Charcot Island, Antarctica (Figure 1, Additional File 1). The individual Macro-048 was obtained from near Ville de Jumelles, St Maur Créteil, and yet is clearly different to other individuals identified as conspecifics from the same sample of moss. Similarly *Acutuncus *sp. was collected from Jubany Station, King George Island, Antarctica. There were clearly two different taxa represented at this one site – one taxon likely to be *Hypsibius*.

Misidentification in these kinds of studies is not surprising as identification can only be carried out under low power in order not to compromise material for subsequent molecular study (we used 400× inverted microscope). Rigorous taxonomic identification requires permanent mounting and viewing under 1000× magnification (see Methods regarding voucher specimens). Thus the limitation of potential misidentification is common to all current molecular studies of tardigrades. Our protocol allows for individual molecular variation to be assessed. Then, where unexpected variation occurs, an informed re-assessment of morphology can be made. To demonstrate this, we took a second sample of substratum from Jubany Station, re-extracted the tardigrades present and mounted multiple individuals to be observed under high power magnification. In this sample *Acutuncus antarcticus *and two undescribed species of *Hypsibius *were identified (data not presented). Furthermore an egg of *Acutuncus antarcticus*, (for which identification is considerably easier due to ornate sculpturing on the case) was taken from the sample and DNA extracted, amplified and sequenced, and the sequence then used to verify which of our samples were *Acutuncus *and which were the unknown (labelled as Hyp/Acut in Figures 1 and 2 to indicate the uncertain identification).

One other study has used individual tardigrades to explore diversity in environmental samples [20]. In this study remarkable unexpected diversity was present, even between morphologically similar groups, which would have been confused if samples had have been pooled. Pooling mixed taxon samples would at best mask the diversity present or, at worst, generate false sequence due to PCR recombination [43]. Indeed, PCR recombination was detected in our study. A blast search of the full sequences of the two aberrant *Echiniscus testudo *18S specimens (EchinT 058 and EchinT 060: Fig 1) closely matched *Echiniscus *sequence previously deposited on GENBANK. However, blasting a small (~100 base) anomalous region of the sequence perfectly matched the yeast *Candida *sp. indicating that the variation in these PCR products was a chimeric artefact generated from two competing templates.

### Utility for population comparisons

Traditionally in population studies a suite of variable markers are developed and used to screen many individuals to explore intraspecific population structure using allele frequency (genic) and genotype frequency (genotypic) approaches. More recently, studies have taken advantage of the genealogical information available in sequence data to investigate contemporary *and *historical population processes [3,44-47]. Our method potentially enables multiple gene sequences to be obtained from individuals allowing genic, genotypic and genealogic analyses [48]. There has only been a single molecular population genetic study of tardigrades [21]. This study provides an important contribution to the field as it indicates that *Echiniscus testudo *has a high migration potential. Importantly though, the data were collected from pooled individuals and are based on a single gene (CO1) which may not accurately reflect the true population structure [49,50]. To thoroughly explore population structure, multiple independent markers are required and intrapopulation variation needs to be evaluated [51]. From our example we can demonstrate intraspecific variation in *Dactylobiotus sp*. samples in all three gene regions examined (the 8 *Dactylobiotus *specimens came from a single portion of moss and are presumed to be conspecifics) and 5 sequence variable alleles at the *Wingless *locus for *Ramazzotius oberhaeuseri *from a single clump of moss.

## Conclusion

We have described a method that allows for extraction of individual meiofaunal specimens from their substratum. Our protocol for assessing between-individual variation is guided by but independent of morphology. We have demonstrated that phylogenetic analyses using data gained from individuals rather than pooled samples allow the detection of hidden diversity and assists in distinguishing true diversity from errors. Sequence data obtained from multiple genes will also have application in improving resolution in population level studies. We are currently employing the techniques described here to conduct large-scale phylogenetic and biogeographic studies of tardigrades.

## Methods

### Sample collection and specimen isolation

Fresh substrata samples of mosses and lichens were collected from walls in St. Maur des Fossés, Paris, France, a type locality for several tardigrade species. Herbarium and frozen samples from sub- and maritime Antarctic islands and from mainland Antarctic nunataks were also examined (See Additional File 1 for details concerning species, locations and genbank accession numbers). Samples were soaked in double-distilled water and transferred to a mini cup blender (Waring). Individual samples of approximately 2 cm^3 ^were homogenised briefly (two short 2 second bursts at the lower speed) then filtered and washed with double-distilled water through a coarse (~1 mm) mesh sieve. The resulting sediment and water were transferred to 1.5 mL micro-centrifuge tubes prepared with OptiPrep™ – Density Gradient Media (Axis-Shield, UK). A layer of 50% OptiPrep™ solution was floated over a 100% solution and the sample added above these layers. The tubes were spun in a bench-top centrifuge (Griffin) at 1000 rpm for 1 minute. The material from the interface between sample water and the 50% OptiPrep™ solution was removed and filtered through a 45 μm mesh sieve, using double-distilled water. The sieve was washed out into a 50 mL Petri dish and viewed under a Wild M5 dissecting microscope. Tardigrades, and eggs where present, were lifted from the Petri dish via Irwin loop (a non-corrosive nickel-chromium wire loop, approx. 200 μm × 500 μm) into a cavity slide filled with double-distilled water. The slide was viewed on a Nikon Diaphot inverted microscope with a 40 to 400× magnification range to identify the tardigrades to genus, species, or type. The individual tardigrades, or eggs, were lifted from the slide via Irwin loop and transferred to 5 μL of double-distilled water in a labelled 0.5 mL microcentrifulge tube and frozen at -80°C.

### Voucher specimens

In any given moss several different taxa were often present. Representatives of each were mounted directly onto slides with de Faure's mounting media [52], and individuals, where possible, were identified to species under high magnification (Olympus BX50 – max. 1000× magnification). These voucher specimens were deposited at the British Antarctic Survey Data Resource Centre.

### DNA isolation and PCR conditions

Individual specimens were subjected to two rounds of freeze-thaw cycling (-80°C to 55°C) in sealed 0.5 mL microcentrifuge tubes to assist in rupturing the cuticle. To each microcentrifuge tube 40 μL of vortexed 5% Chelex 100 (Biorad) was added and the specimens incubated at 99°C for 20 min. Samples were centrifuged for 1 min at maximum speed in a microcentrifuge (Eppendorf) and stored at -20°C. A blank (5 μL H_2_O, 40 μL 5% chelex) was treated in the same way and used as a negative control in PCR.

We attempted to quantify the amount of DNA present in the extract using a PicoGreen™ (Molecular Probes) assay. We optimised the assay for 10 μL total volume (5 μL of sample) to minimise background fluorescence with a standard ranging from 1 ng μL^-1 ^to 0.00001 ng μL^-1 ^to be detected on a Q-PCR thermo-cycler (Stratagene). The standard curve detected DNA fluorescence to 0.0005 ng μL^-1 ^after which the relationship between fluorescence and DNA concentration was no longer linear. Our samples did not differ from background fluorescence (<0.0001 ng μL^-1^).

To increase the yield of PCR products we labelled each primer with an M13 tail, the forward primers with M13 REV and the reverse primers with M13 -21 [53]. Primer details are given in Table 1.

We amplified the nuclear "multi-copy" gene *18S *rDNA (18S) and the mitochondrial (thus high copy number) *cytochrome c oxidase subunit *1 (*CO1*) gene for which there were data available on GENBANK to verify that the products we were amplifying were from the target organisms as opposed to contamination from airborne substrata, gut contents or commensal organisms associated with the tardigrade cuticle [17]. For *18S *we used the primers SSU 1F and modified SSU 82R [54,55] to amplify approximately 1800 bases in 10 μL volumes containing 16 μM ammonium sulphate, 68 mM Tris-HCl (pH 8), 10 mM β-mercaptoethanol, 5% bovine serum albumin 10 mg/mL (Sigma), 3 mM magnesium chloride, 200 μM each dNTP, 0.5 μM each primer, 0.5 units of *Taq *DNA Polymerase (Bioline), and 1 μL template DNA. Cycling conditions were 94°C for 2 min followed by 35 cycles of 94°C for 1 min, 60°C for 30 sec and 72°C for 1 min. This was followed by a 4 min extension at 72°C.

Using 2 μL of this reaction mixture we conducted three separate reactions to amplify three overlapping fragments of the gene using the primers SSU 1F/SSU 26R, SSU 22F/SSU 13R and SSU 26F/SSU 82R [55] (see Table 1). All three fragments were amplified in 40 μL volumes using the above conditions with the exception of magnesium chloride concentration, which was lowered to 2 mM, and annealing temperature, which was raised to 65°C, to increase specificity.

The mitochondrial *CO1 *gene was amplified by hemi-nested PCR. The first amplification was with the primers LCO_1490 [56] and mtD9 [57] in 10 μL volumes containing 16 μM ammonium sulphate, 68 mM Tris-HCl (pH 8), 10 mM β-mercaptoethanol, 5% bovine serum albumin 10 mg/mL (Sigma), 2 mM magnesium chloride, 200 μM each dNTP, 0.5 μM each primer, 0.5 units of *Taq *DNA Polymerase (Bioline), and 1 μL template DNA. Cycling conditions were 94°C for 2 min followed by 35 cycles of 94°C for 30 sec, 45°C for 30 sec and 72°C for 45 sec. This was followed by a 4 min extension at 72°C.

The second amplification was with the primers LCO_1490 and HCO_2198 [56]. Reaction conditions were in 40 μL volumes using the above concentrations. Cycling conditions were as above but with annealing temperature raised to 50°C.

A similar nested amplification strategy was applied to the genes *Wingless*, *Alpha Spectrin *and *Elongation Factor 1 alpha*, using the initial PCR cycling conditions suggested by Regier [58]. Amplifications were conducted in 10 μL (first amplification) and 40 μL (second amplification) reactions using reaction mix concentrations as above. The cycling conditions for the first amplification were 94°C for 2 min followed by 24 cycles starting 94°C 30 sec, 56°C 30 sec, 72°C 1 min but decreasing annealing temperature from 56°C to 45°C by 0.4°C each cycle and increasing extension time by 2 seconds each cycle. This was followed by a further 12 cycles of 94°C 30 sec, 45°C 30 sec 72°C 2 min increasing the extension time by 3 seconds each cycle. The reaction was terminated with a 4 min extension at 72°C. The second amplification used 2 μL of the first reaction as template but used more conventional cycling conditions of 94°C for 2 min followed by 35 cycles of 94°C for 30 sec, 45°C for 30 sec and 72°C for 45 sec. This was followed by a 4 min extension at 72°C. Amplified products were sequenced by Macrogen inc. Seoul, Korea. All sample information including GenBank accession numbers are available as an additional file.

### Analyses

Sequence trace files were base-called and aligned using Codoncode Aligner V1.6.3 (CodonCode Corp). Coding genes were checked for open reading frame and blast searched (tblastx) to assess gene homology. Where highly divergent nucleotide sequences proved difficult to align, sequences were converted to amino acids and aligned by eye and back translated to nucleotide in SE-AL [59]. Ambiguous alignment regions were excised from the analyses.

To determine an appropriate model of sequence evolution we used the iterative optimisation approach suggested by Swofford [60] and validated by Sullivan [61]. The data was used to construct a neighbour joining (NJ) tree in PAUP*4 10b [62] from which the likelihood parameters were estimated and used in a heuristic likelihood tree search (NJ starting tree, TBR). After a minute the search was stopped and likelihood parameters were estimated from the new tree. These parameters were used to conduct another likelihood search that was again stopped after a minute. This procedure was repeated until the likelihood scores stabilised. Once parameters were optimised for the full model, we systematically reduced the number of parameters to see if a simpler model could be used to describe the patterns of evolution without adversely affecting the overall likelihood scores. Using the appropriate model (GTR+I+Γ) we conducted heuristic searches with 100 random starting addition sequences using maximum likelihood option in PAUP*. We conducted 1000 bootstrap replicates (1 random addition sequence) to assess the strength of each phylogenetic inference.

## Competing interests

The authors declare that they have no competing interests.

## Authors' contributions

CJS performed all molecular procedures and analyses and prepared the initial manuscript. PC collected samples from Antarctic environs, contributed to the project design and the manuscript. KL initiated and led the project, contributed the project design, and contributed to the text. SJM collected samples from France, conducted all isolation and taxonomic identification and contributed to the manuscript.

## Supplementary Material

Additional File 1 **Sample information**. Sample information provided includes taxonomic identification, location, sample type and GenBank accession numbers. Click here for file
